# Dried Porous Biomaterials from Mealworm Protein Gels: Proof of Concept and Impact of Drying Method on Structural Properties and Zinc Retention

**DOI:** 10.3390/gels10040275

**Published:** 2024-04-18

**Authors:** Martina Klost, Claudia Keil, Pavel Gurikov

**Affiliations:** 1 Faculty III Process Sciences, Institute for Food Technology and Food Chemistry, Department of Food Technology and Food Material Science, Technische Universität Berlin, Straße des 17. Juni 135, 10623 Berlin, Germany; martina.klost@tu-berlin.de; 2 Faculty III Process Sciences, Institute of Food Technology and Food Chemistry, Department of Food Chemistry and Toxicology, Technische Universität Berlin, Straße des 17. Juni 135, 10623 Berlin, Germany; c.keil@tu-berlin.de; 3 Laboratory for Development and Modelling of Novel Nanoporous Materials, Hamburg University of Technology, Eißendorfer Straße 38, 21073 Hamburg, Germany; 4 aerogel-it GmbH, Albert-Einstein-Str. 1, 49076 Osnabrück, Germany

**Keywords:** aerogel, lyophilized hydrogel, biobased, *Tenebrio molitor*, zinc

## Abstract

Dried porous materials can be found in a wide range of applications. So far, they are mostly prepared from inorganic or indigestible raw materials. The aim of the presented study was to provide a proof of concept for (a) the suitability of mealworm protein gels to be turned into dried porous biomaterials by either a combination of solvent exchange and supercritical drying to obtain aerogels or by lyophilization to obtain lyophilized hydrogels and (b) the suitability of either drying method to retain trace elements such as zinc in the gels throughout the drying process. Hydrogels were prepared from mealworm protein, subsequently dried using either method, and characterized via FT-IR, BET volume, and high-resolution scanning electron microscopy. Retention of zinc was evaluated via energy-dispersive X-ray spectroscopy. Results showed that both drying methods were suitable for obtaining dried porous biomaterials and that the drying method mainly influenced the overall surface area and pore hydrophobicity but not the secondary structure of the proteins in the gels or their zinc content after drying. Therefore, a first proof of concept for utilizing mealworm protein hydrogels as a base for dried porous biomaterials was successful and elucidated the potential of these materials as future sustainable alternatives to more conventional dried porous materials.

## 1. Introduction

Dried porous materials such as aerogels can be found in applications ranging from aerospace and construction to various life and food science branches. With porosity (at the micro (<2 nm), meso- (2–50 nm), and macrolevels (>50 nm), high porosity (up to 99.98%) and large surface areas (up to 1500 m^2^/g) aerogels can be applied in various areas [1,2]. Bio-based aerogels made from either biopolymer materials (such as cellulose, chitosan, and alginate) [3,4] or from renewable side streams (banana peel, apple pomace, and coffee powders) [5,6] are highly valued in life- and food science applications. They may serve as carriers for bioactive compounds, functional ingredients, or micronutrients. For example, they can be used as carrier systems for trace elements requiring gastrointestinal compatibility [7,8,9]. One example for such trace elements is zinc of which about 17% of the world’s population has a deficiency. As a consequence, it is critical to find a way to provide zinc in a food or supplement format that is easily accessible [10,11].

Research efforts towards the production of tailor-made aerogels from bio-based raw materials have vastly increased over the past decade [12]. While our estimations show that over a thousand peer-reviewed publications appear annually on biopolymer aerogels, most of these efforts focus on cellulose and other enzymatically inaccessible carbohydrates as building blocks for porous gels. However, the indigestibility of polysaccharide-based aerogels may not be appropriate in all applications. This has led to a growing number of studies focusing on protein aerogels derived from various sources such as milk, eggs, and different plants [13,14] (and references therein). However, this research branch still only accounts for approximately 3.5% of all research on biopolymer-based aerogels. When considering protein sources, not only should their techno-functional properties be considered, but their environmental impact should also be considered [6,15]. In this context, insects such as mealworms (*Tenebrio molitor*) are considered to be promising because they require only a little water and can be reared on organic waste [16]. Moreover, mealworm is an EFSA-approved raw material for food applications [17,18]. However, research on the gelation of mealworm protein is only just emerging [19], and, to our knowledge, so far, there are no reports on dried porous biomaterials prepared from mealworm protein gels. Therefore, the aim of the presented study was to provide a first proof of concept for the suitability of mealworm protein hydrogels to be turned into dried porous biomaterials and to investigate whether these gels could retain added zinc as a model trace element throughout the fabrication process.

## 2. Results and Discussion

### 2.1. Characterization of Aerogels and Lyophilized Hydrogels

**Figure 1:**
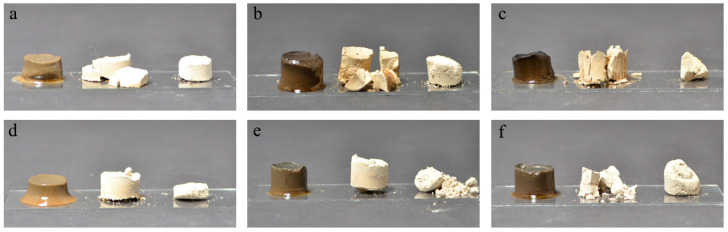
Photographs of HG (**left**), LHG (**center**), and AG (**right**) from mealworm protein gels prepared in distilled water at pH 3.5 (**a**), 5.5 (**b**), 7.5 (**c**), and 0.3 M ZnSO_4_ solution at pH 3.5 (**d**), 5.5 (**e**), and 7.5 (**f**).

In this section, we will mainly focus on the characterization and comparison of dried porous biomaterials such as aerogels (AG) and lyophilized hydrogels (LHG) from mealworm protein hydrogels (HG) prepared in distilled water. Discussion on the coordination of zinc with protein will additionally compare results from samples prepared in distilled water to those from samples prepared with zinc. As previously shown by Klost et al., mealworm protein could form heat-induced hydrogels (HG) with and without the addition of ZnSO_4_ [19]. At the selected protein concentration (13.4%) and pH values (3.5, 5.5, and 7.5) in the present study, the hydrogels were self-supporting—more so at pH 5.5 and 7.5 compared to pH 3.5—and could be turned into dried gels by both solvent exchange or lyophilization, leading to aerogels (AG) and lyophilized hydrogels (LHG) respectively (Figure 1). The brittleness of some of the dried gels (as shown in Figure 1) may be related to the nature of the interactions stabilizing the hydrogels (i.e., hydrogen bonds, hydrophobic interactions, and some ionic or covalent bonds, especially if the gels contain zinc [19]) as well as the distribution of these interactions within and between aggregates in the gel.

The microstructure is of utmost relevance to future applications in dried porous materials. Therefore, the AG and LHG were characterized and compared with regard to the impact of the drying method on the molecular and microscopic levels.

**Figure 2:**
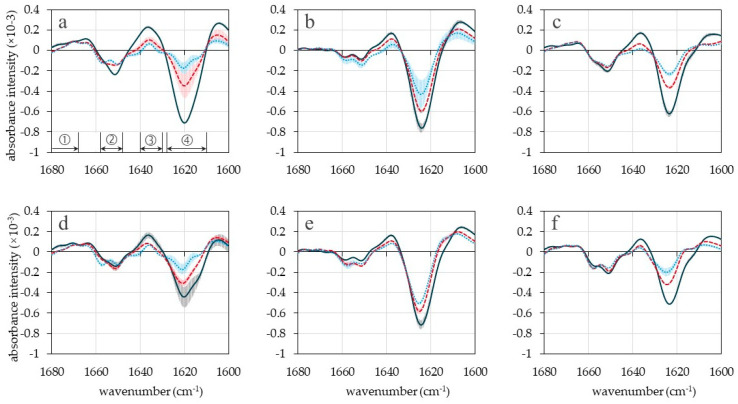
Second derivate spectrum from FT-IR measurements at wavenumbers representing the amide I band. (**a**) HG (**b**) AG (**c**) LHG prepared in distilled water; (**d**) HG (**e**) AG (**f**) LHG prepared in 0.3 M ZnSO_4_ solution. Black lines: pH 3.5, red lines: pH 5.5, and blue lines: pH 7.5. Peaks in zone 1 represent antiparallel intramolecular β-sheets, peaks in zone 2 represent α-helices, peaks in zone 3 represent parallel intramolecular β-sheets and peaks in zone 4 represent intermolecular β-sheets. Error bars represent deviations between individual measurements of the same sample.

Characterization of AG and LHG at the molecular level and comparison to the HG was carried out via FT-IR (Figure 2). FT-IR spectra of HG are dominated by intermolecular β-sheet structures (approx. 1620 cm^−^^1^) that formed during the heating process [20]. Drying leads to a shift in the wavenumber of intermolecular β-sheets from approximately 1620 cm^−^^1^ (Figure 2a,d) toward approximately 1624 cm^−1^ (Figure 2b,c,e,f), indicating a slight weakening of the involved intermolecular hydrogen bonds [21] independent of drying method and addition of zinc. Observed differences in peak intensity, e.g., depending on pH value or drying method in Figure 2, cannot be attributed to differences between samples because Figure 2 shows the second derivative of the spectrum and, therefore, only allows for qualitative evaluation.

Besides obtaining information on secondary structures of the protein, FT-IR spectroscopy can give some information on the coordination of metal ions with proteins. The literature implies that oxygen, nitrogen, and sulfur donors from amino acid side chains limit the zinc coordination environment in proteins under physiological conditions. Changes in the number of ligands available for the metal ions, the ideal geometry of the protein ligands, or the number of interactions between the secondary coordination sphere and the global protein structure can change zinc’s affinity to the protein [22,23]. Since we are looking at heat-treated and, therefore, non-native proteins in our study, the physiological affinity of the proteins to the zinc will most likely be altered. With regard to the coordination of metal ions with various proteins, the literature indicates an impact of these cations on the νCOO^−^ symmetric (1430–1360 cm^−^^1^) and asymmetric (1580–1560 cm^−^^1^) stretching vibrations, e.g., [24,25]. Shifts in the frequencies of the respective bands and their relation to each other may, in turn, allow for the interpretation of the coordination mode of the metal ions [24,26]. Since the νCOO^−^_asym_ band overlaps with the amid II band, it cannot easily be distinguished in our study. For hydrogels, we found the wavenumbers of the νCOO^−^_sym_ band to increase slightly with the addition of ZnSO_4_ at pH 5.5 and 7.5 and to decrease slightly at pH 3.5. Compared to shifts shown in the literature for various metal ions with Lactobacillus kefir S-layers [25], these shifts are very minor. Moreover, without information on the wavenumber of the νCOO^−^_asym_ band, they cannot be related to any coordination mechanism. Consequently, future research should be conducted under conditions where exchangeable protons in the mealworm protein are completely deuterated [24] so that the νCOO^−^_asym_ band can be evaluated more easily. 

Regarding the coordination of zinc with sulfur donors, in our study, we did not consider bands corresponding to sulfur groups as a distinction between Zn-sidechain interactions and the effect of the utilized salt could not be sufficiently distinguished.

**Figure 3:**
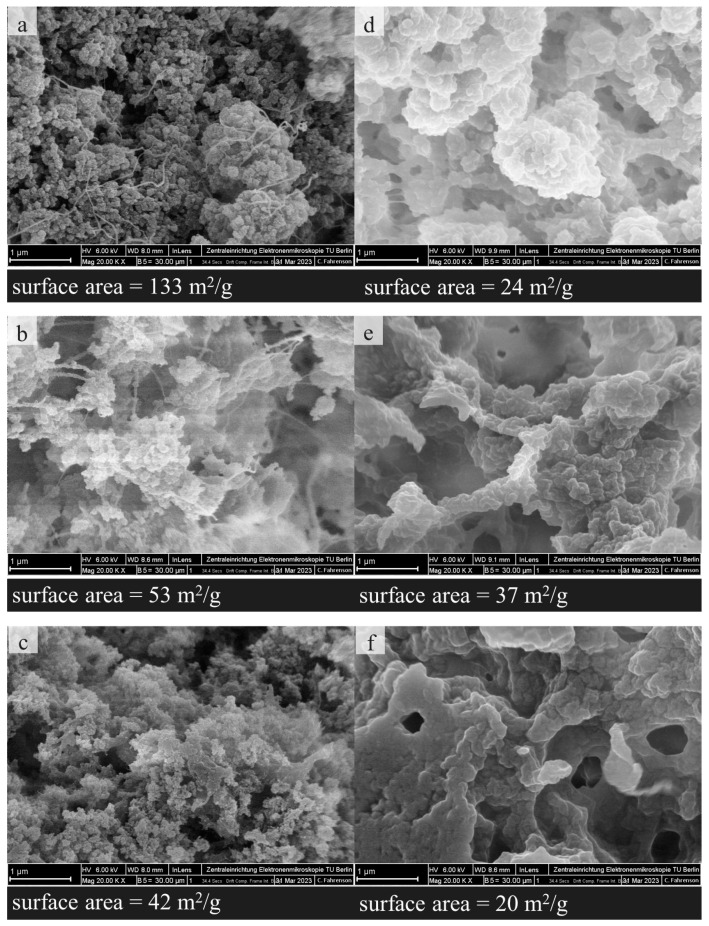
SEM pictures and surface areas of AG and LHG (both zinc-free) at 20,000× magnification at pH 3.5, 5.5, and 7.5, respectively. (**a**) AG-pH 3.5, (**b**) AG-pH 5.5, (**c**) AG-pH 7.5, (**d**) LHG-pH 3.5, (**e**) LHG-pH 5.5, and (**f**) LHG-pH 7.5.

Determination of the BET surface area was used to investigate structural properties at the microscopic level (Figure 3; see Supplementary Figure S2 for adsorption isotherms). With regard to the AG, we found an increase in the surface area with decreasing pH value (cf. 133, 53, and 42 m^2^/g for AG prepared at pH 3.5, 5.5. and 7.5, respectively; Figure 3a–c). This indicates a higher porosity of the corresponding samples, at least in the mesoporous range [27]. A similar behavior of the specific surface area with respect to the pH increase has been reported by Andlinger et al. [13] for potato protein aerogels. Differences in the protein conformation conventionally explain the drop in the surface area: compacted globular structures are present in the gelling solution at a pH close to the isoelectric point. The resulting gels and corresponding aerogels thus demonstrate a more granular structure with a lower specific surface compared to the gels obtained under acid or basic conditions. LHG did not show a distinct influence of pH (24, 37, and 20 m^2^/g for LHG prepared at 3.5, 5.5, and 7.5, respectively, Figure 3d–f).

The BET constant C is also distinctly higher for AG than for LHG (81 vs. 8 for AG and LHG at pH 3.5, respectively). As higher values for C indicate a more polar surface [28], we can conclude that solvent exchange-based drying preserves the polar surface of a HG to a greater extent while freezing in liquid nitrogen followed by lyophilization leads to more hydrophobic pores in the corresponding LHG. These findings are supported by high-resolution SEM micrographs recorded at 20,000× magnification. In agreement with results from surface area measurements, AG (Figure 3a–c) shows finer, more porous structures compared to the corresponding LHG (Figure 3d–f).

### 2.2. Retention of Zinc during Drying of Mealworm Protein Hydrogels

**Figure 4:**
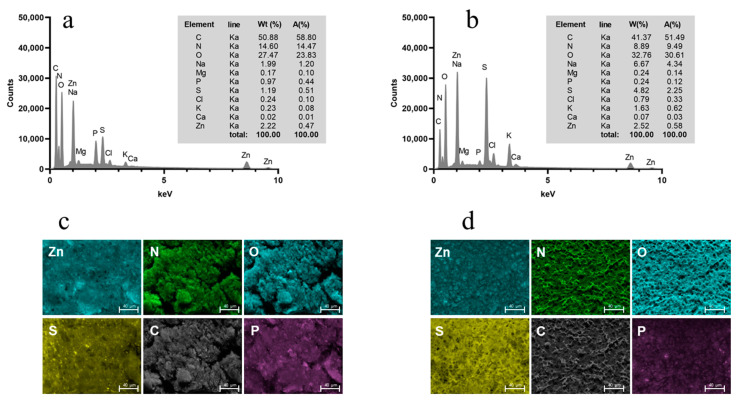
EDX spectra of AG (**a**) and LHG (**b**) prepared with 0.3 M ZnSO_4_. The inset table shows the results of the semi-quantitative standardless EDX analysis. EDX maps (k line of x-ray emission) of the spatial distributions of Zn, N, O, S, C, and P in the AG (**c**) and LHG (**d**). Scale bars = 40 μm.

With regard to the future utilization of AG and/or LHG as possible delivery systems for trace elements in biomedical applications, this proof of concept also aimed to determine if zinc added in the course of HG fabrication would be retained in the samples during the drying procedure. With regard to a potential loss of Zn^2+^, it can be assumed that the zinc should be retained in the sample during freeze drying as it is not volatile. However, with regard to the solvent exchange processes during AG preparation, zinc may be washed from the samples, as has already been shown for zinc-alginate gels in the past [29]. To this purpose, we investigated AG and LHG using energy-dispersive X-ray spectroscopy to determine their elemental composition. Figure 4 shows the corresponding EDX spectra and EDX maps for samples prepared at pH 5.5 as an example. 

If AG and LHG were produced without adding ZnSO_4_, no zinc could be determined in the corresponding EDX spectra (Supplementary Figure S1). In contrast, all AG and LHG that had been produced with the addition of ZnSO_4_ retained some of the zinc throughout either drying process. This is, to some extent, in agreement with previous findings, where zinc was shown to decrease the gel solubility of mealworm protein hydrogels in various aqueous solvents, thus indicating an incorporation of zinc into the protein gel network structure. In that study, the decrease in gel solubility was related to the additional occurrence of some ionic or covalent bonds [19].

## 3. Conclusions

The presented study showed that mealworm protein hydrogels form dried porous bio-materials when processed into aerogels by supercritical CO_2_ drying, or lyophilization to obtain lyophilized hydrogels. With regard to future utilization in biomedical, life science, and food applications, we could demonstrate that added zinc could be retained throughout drying by lyophilization or solvent exchange followed by supercritical CO_2_ drying. From a mechanistic point of view, future research should aim at further investigation of structural changes on the molecular level and investigation of effects causing the shift towards more hydrophobic pores in lyophilized hydrogels compared to the corresponding aerogels. In addition, the influence of this difference in pore hydrophobicity on techno-functional properties such as water uptake, swelling rate, and solubility should be investigated with regard to future applications. In principle, zinc-binding protein domains and zinc-binding sequence motifs are found in all domains of life. As mentioned above, there are various limiting factors to the coordination environment of zinc in proteins. These may be related to the availability of ligands as well as to protein conformation [22,23]. The zinc proteome accounts for about 9% of the total proteome in eukaryotes. For example, in the fruit fly *Drosophila melanogaster,* the zinc proteome represents about 10.2% of the entire proteome [30]. Metal-protein interactions under cell/tissue homeostasis conditions involve a dynamic coordination environment that includes mechanisms for metal dissociation and association over a timescale of seconds to years. However, zinc’s transfer into proteins differs considerably when administered under chemical/technological conditions, as in our approach [31,32]. So, further research is needed to understand the zinc coordination spheres in the mealworm HG and possible changes in the course of the conversion to AG/LHG protein structures. 

To counteract the brittleness of some of the AG and LHG, further experiments should additionally focus on increasing the gel-stability. To this regard, crosslinking with transglutaminase or protein modification with sodium triphosphate are potential options. The former leads to iso-peptide bonds between glutamine and lysine residues, while the latter promotes the formation of salt bridges by zinc ions [33].

Drying is considered to be the most critical step in the aerogel production process since it preserves the three-dimensional pore structure that results in unique material properties. Both the supercritical CO_2_ and freeze-drying methods are widely used to produce aerogels, e.g., [34,35,36]. In recent years, ambient pressure, microwave, and vacuum drying have also gained popularity [4,37]. The ability to tailor bio-based sustainable aerogel properties to meet application requirements requires a thorough understanding of how different processing parameters affect microstructure.

With regard to possible applications of mealworm protein gels in the biomedical, life-science, and food sectors, future research should additionally focus on the investigation of the release of retained trace elements such as zinc. 

Despite the necessity for future research toward a complete comprehension of these novel dried porous biomaterials from mealworm gels, the proof of concept in this study elucidated their potential as sustainable alternatives to more conventional dried porous materials.

## 4. Materials and Methods

### 4.1. Materials

Living mealworms were purchased from ENTAVA (Roggentin, Germany), frozen in liquid nitrogen, and subsequently lyophilized. Mealworm proteins were extracted according to Klost et al. [19]. The protein content was 65.4% (derived from nitrogen content according to Dumas (DUMATHERM DT, C. Gerhardt, Königswinter, Germany), protein factor 5.60 [37]). Hexane, HCl, NaOH, ethanol, and ZnSO_4_ were purchased from Carl Roth (Karlsruhe, Germany) or VWR (Darmstadt, Germany) and were of analytical grade. Denatured ethanol and CO_2_ for drying were obtained from Carl Roth (Karlsruhe, Germany) and Nippon Gases Deutschland GmbH (Hürth, Germany), respectively.

### 4.2. Preparation of Mealworm Protein Hydrogels, Lyophilized Hydrogels and Aerogels

Solutions with 13.4% protein content were prepared in distilled water, according to Klost et al. [19]. pH was adjusted to pH 3.5, 5.5 or 7.5 with NaOH and HCl, respectively. Gels containing zinc gels were prepared with 0.3 M ZnSO_4_ instead of water. Solutions were subsequently poured into plastic cylinders (14 mm diameter, 14 mm height), covered with a Petri dish to avoid evaporation, and heat-induced gelation was induced by placing the samples into a drying cabinet at 90 °C for 30 min. One batch of the hydrogel (HG) samples was turned into alcogels by treatment with a large excess of 100% ethanol (approx. 1:10 gel:EtOH *v*/*v*). After the solvent exchange, the alcogels were dried with supercritical carbon dioxide at 35 °C and 100 bar for 2 h to obtain aerogels (AG). Another batch of HG samples was produced for characterization of the HG and subsequent freeze drying to obtain the lyophilized hydrogels (LHG). To this purpose, HG was frozen in liquid nitrogen and subsequently lyophilized (Beta 1–8 LSCplus, Christ Gefriertrocknungsanlagen, Osterode am Harz, Germany). As the study was intended only as a proof-of-concept, no replicates were prepared.

### 4.3. Fourier Transform Infrared Spectroscopy

FT-IR spectra of AG, HG, and LHG were recorded at room temperature in the range from 4000 to 800 cm^−1^ (Bruker Optic GmbH, Karlsruhe, Germany) equipped with a liquid nitrogen-cooled mercury-cadmium-telluride detector. Measurements were carried out at least eight times from the same sample. The second derivative within the amide I band (1680 to 1600 cm^−1^) was calculated to investigate changes to the secondary structure of the protein.

### 4.4. Determination of the Specific Surface Area

Determination of the specific surface areas of AG and LHG was carried out by low-temperature N_2_ adsorption analysis (NOVA 4000e, Quantachrome Instrument. Anton Paar, Graz, Austria) using the Brunauer–Emmett–Teller (BET) method [6].

### 4.5. High-Resolution Scanning Electron Microscopy and X-ray Spectra

Gold sputtering and high-resolution scanning electron microscopy (HR-SEM) analysis of AG and LHG samples was done at the Center for Electron Microscopy (ZELMI), Technische Universität Berlin, Germany (Microscope ZEISS GeminiSEM500 Nano VP with an in-lens detector (Carl Zeiss Microscopy GmbH, Jena, Germany)). Energy-dispersive X-ray spectra were also recorded at the Center for Electron Microscopy (ZELMI), Technische Universität Berlin, Germany, using S-2700 scanning electron microscope (Hitachi, Tokyo, Japan) with SDD-detector with Si3N4-window (remX GmbH (Bruchsal, Germany)).

## Data Availability

The data presented in this study are available upon request from the corresponding author.

## Supplementary Materials

The following supporting information can be downloaded at: https://www.mdpi.com/article/10.3390/gels10040275/s1, Figure S1: EDX spectra of all samples prepared with and without 0.3 M ZnSO_4_; Figure S2: Nitrogen adsorption isotherms of AG (a) and LHG (b) at pH 3.5, 5.5 and 7.5 respectively.
