# Supplementary material for: Dried Porous Biomaterials from Mealworm Protein Gels: Proof of Concept and Impact of Drying Method on Structural Properties and Zinc Retention
Source: Gels. 2024 Apr 18;10(4):275. doi: 10.3390/gels10040275 (PMC11049402; doi:10.3390/gels10040275)
Supplement: Supplementary file 1 [file gels-10-00275-s001.zip › gels-2912051-supplementary.pdf]

Supplementary Materials

Dried porous biomaterials from mealworm protein gels: Proof of concept and impact of drying method on structural properties and zinc retention

Martina Klost [1] (https://orcid.org/0000-0001-7131-6406), Claudia Keil [2] (https://orcid.org/0000-0003-0317-0905) and Pavel Gurikov [3,4]* (https://orcid.org/0000-0003-0598-243X)

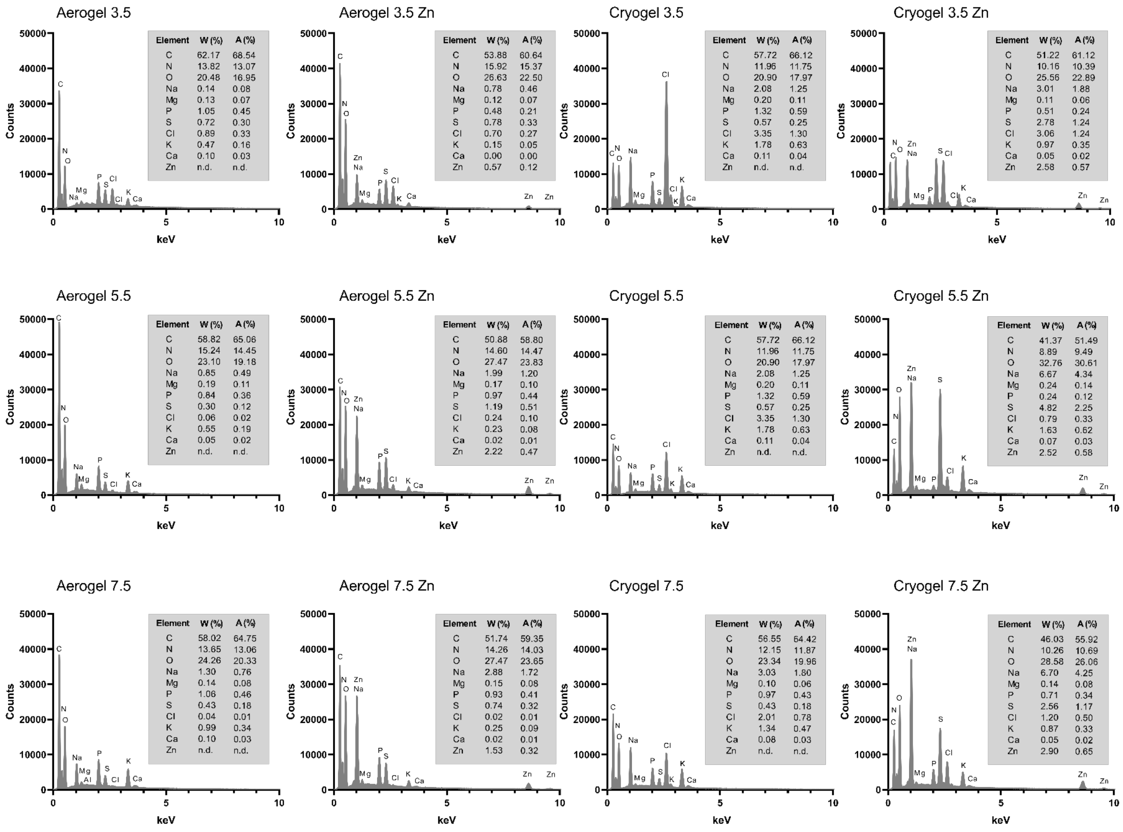

Figure S1. EDX spectra of all samples prepared with and without 0.3 M $ZnSO_4$. The inset table shows results of semi-quantitative standardless EDX analysis.

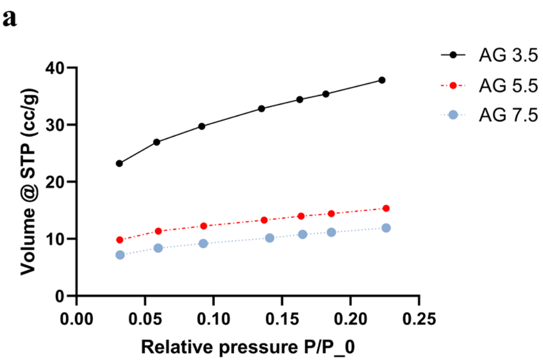

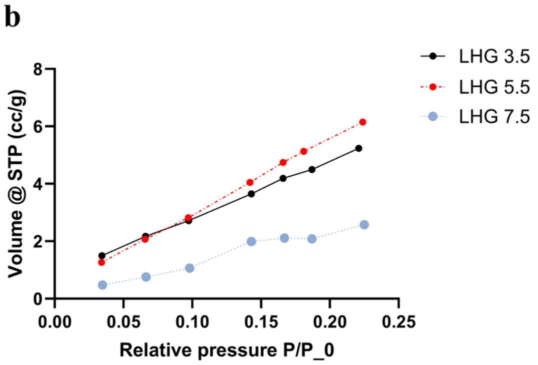

Figure S2. Nitrogen adsorption isotherms of AG (a) and LHG (b) at pH 3.5, 5.5 and 7.5 respectively.
